# Supplementary material for: Resolving the full spectrum of human genome variation using Linked-Reads
Source: Genome Res. 2019 Apr;29(4):635–45. doi: 10.1101/gr.234443.118 (PMC6442396; doi:10.1101/gr.234443.118)
Supplement: Supplemental Material [file supp_gr.234443.118_Supplemental_longranger-2.2.zip › longranger-2.2.tar.bz2!/lr2.2-public/lib/stan/cmdstan/stan/doc/api/html/dense__e__nuts_8hpp_source.html]

Stan: src/stan/mcmc/hmc/nuts/dense\_e\_nuts.hpp Source File


|  |  |
| --- | --- |
|  | Stan  2.5.0  probability, sampling & optimization |


- Main Page
- Related Pages
- Namespaces
- Classes
- Files

- File List
- File Members

All Classes Namespaces Files Functions Variables Typedefs Enumerations Enumerator Friends Macros Pages

- src
- stan
- mcmc
- hmc
- nuts

dense\_e\_nuts.hpp

Go to the documentation of this file.

1 #ifndef STAN\_\_MCMC\_\_DENSE\_\_E\_\_NUTS\_\_BETA

2 #define STAN\_\_MCMC\_\_DENSE\_\_E\_\_NUTS\_\_BETA

3

4 #include <stan/mcmc/hmc/nuts/base\_nuts.hpp>

5 #include <stan/mcmc/hmc/hamiltonians/dense\_e\_point.hpp>

6 #include <stan/mcmc/hmc/hamiltonians/dense\_e\_metric.hpp>

7 #include <stan/mcmc/hmc/integrators/expl\_leapfrog.hpp>

8

9 namespace stan {

10

11  namespace mcmc {

12

13  // The No-U-Turn Sampler (NUTS) on a

14  // Euclidean manifold with dense metric

15

16  template <typename M, class BaseRNG>

17  class dense\_e\_nuts: public base\_nuts<M,

18  dense\_e\_point,

19  dense\_e\_metric,

20  expl\_leapfrog,

21  BaseRNG> {

22

23  public:

24

25  dense\_e\_nuts(M &m, BaseRNG& rng, std::ostream\* o = &std::cout, std::ostream\* e = 0):

26  base\_nuts<M, dense\_e\_point, dense\_e\_metric, expl\_leapfrog, BaseRNG>(m, rng, o, e)

27  { this->name\_ = "NUTS with a dense Euclidean metric"; }

28

29  // Note that the points don't need to be swapped

30  // here since start.mInv = finish.mInv

31  bool compute\_criterion(ps\_point& start,

32  dense\_e\_point& finish,

33  Eigen::VectorXd& rho) {

34  return finish.p.transpose() \* finish.mInv \* (rho - finish.p) > 0

35  && start.p.transpose() \* finish.mInv \* (rho - start.p) > 0;

36  }

37

38  };

39

40  } // mcmc

41

42 } // stan

43

44 #endif

stan::mcmc::base\_mcmc::name\_

std::string name\_

**Definition:** base\_mcmc.hpp:38

stan::mcmc::dense\_e\_nuts::compute\_criterion

bool compute\_criterion(ps\_point &start, dense\_e\_point &finish, Eigen::VectorXd &rho)

**Definition:** dense\_e\_nuts.hpp:31

stan::mcmc::ps\_point

**Definition:** ps\_point.hpp:16

stan::mcmc::dense\_e\_metric

**Definition:** dense\_e\_metric.hpp:20

stan::mcmc::base\_nuts

**Definition:** base\_nuts.hpp:32

stan::mcmc::dense\_e\_nuts::dense\_e\_nuts

dense\_e\_nuts(M &m, BaseRNG &rng, std::ostream \*o=&std::cout, std::ostream \*e=0)

**Definition:** dense\_e\_nuts.hpp:25

stan::mcmc::ps\_point::p

Eigen::VectorXd p

**Definition:** ps\_point.hpp:48

dense\_e\_metric.hpp

stan::mcmc::dense\_e\_point::mInv

Eigen::MatrixXd mInv

**Definition:** dense\_e\_point.hpp:18

dense\_e\_point.hpp

stan::mcmc::expl\_leapfrog

**Definition:** expl\_leapfrog.hpp:12

expl\_leapfrog.hpp

stan::math::e

double e()

Return the base of the natural logarithm.

**Definition:** constants.hpp:86

stan::mcmc::dense\_e\_point

**Definition:** dense\_e\_point.hpp:12

base\_nuts.hpp

stan::mcmc::dense\_e\_nuts

**Definition:** dense\_e\_nuts.hpp:17


---

[ Stan Home Page ]

*© 2011–2014,
Stan Development Team.*
